# Supplementary material for: The explosive value of the networks
Source: Sci Rep. 2023 Jan 19;13:1037. doi: 10.1038/s41598-022-26961-x (PMC9852569; doi:10.1038/s41598-022-26961-x)
Supplement: Supplementary file 1 — Supplementary Information. [file 41598_2022_26961_MOESM1_ESM.pdf]

Supplementary Informations to "The explosive value of the networks"

Antonio Scala[1,2,*] **and Marco Delmastro**[2,+]

[1]CNR-ISC, Applico Lab, Roma, 00185, Italy
[2]Centro Ricerche Enrico Fermi, Roma, 00184, Italy
[*]antonio.scala@cnr.it
[+]marco.delmastro@cref.it; ORCID: 0000-0002-8527-3117

ABSTRACT

In these Supplementary Information we provide details: on the empirical growth analysis of network companies (sec. **S1**) and the techniques used to assess model selection and evaluate their prediction power (sec. **S2**).

S1 Network Value growths

Let's consider a system of N users. When linking together such users, we can define different possible "network values" V[1]:

- Sarnoff : $V \propto N$, corresponding to the possibility of broadcasting a message to all the N users[2].
- Odlyzko-Tilly : $V \propto N \ln N$, corresponding to assuming that each users has a utility proportional to $\ln N$ (i.e. the probability that his k^{th} connection is useful to him is proportional to $1/k$)[3].
- Metcalfe : $V \propto N^2$, corresponding to giving a constant value to all the possible links among the N units[4].
- Nivi : $V \propto N^\gamma$, a phenomenological power-law growth introduced in[5].
- Reed : $V \propto 2^N$, corresponding to giving a constant value to all the possible subsets of N units[6].

Thus, Sarnoff[2] relates the value to the possibility of reaching all the users of the network at once, like in the old broadcasting media (radio, television). On the other hand, Metcalfe relates the value to the number of possible connections among users of the system[4]; it was originally presented in terms of "compatible communicating devices" (i.e. the concept is born after the introduction of telephone networks) and then applied to describe Ethernet connections. Since it is unreasonable to assume that all the possible connections are useful to each user (i.e the number of "useful" connection is limited at least by bounded rationality constraints such as Dunbar's number[7]), Metcalfe himself proposed that the number of useful connections could eventually saturate[4], leading for large N to a linear growth (like Sarnoff) with a large prefactor. On the same footing, inspired by search-engine, Odlyzko-Tilly[8] models the value per user for finding useful contacts in the network with a Zipf statistics; however, in this case, Odlyzko-Tilly should be modified as $V \propto N \ln M$ where M is the number of objects that users can retrieve. Reed related the value of a network to the possibility of forming groups (like on social media). Common-sense suggest that Reed's laws, as stated, is incorrect: since every new users doubles the value of a network, no matter how small the prefactor, it would quickly reach the value of the world economy. Notice that, to explain network value growths stronger than Metcalfe but not as steep as Reed's 2^N, Nivi proposed empirically to use power laws[5].

To determine which law fits better our data on the network values, we apply non-linear least square minimization of the following functional forms

$V = aN$	Sarnoff
$V = aN \ln N$	Odlyzko-Tilly
$V = aN^2$	Metcalfe
$V = aN^\gamma$	Nivi
$V = ae^{\rho N}$	generalised Reed

that capture the scaling of network values respectively for Sarnoff's, Otziko-Tilly's, Metcalfe's, Nivi's and generalised Reed's model. In Table S1 we report the coefficients of determination R^2 of the regression for the US dataset to the various growth laws, while in Table S2 we report the R^2 for the regressions on Meta's revenues per geographical area.

S2 Model selection and prediction power

Nested Models selection

The goodness of fit of two competing models can be compared based on the ratio of their likelihoods or, equivalently, on the difference of their log-likelihood. The likelihood-ratio test, also known as Wilks test, requires that the models to compare are nested, i.e. that one model can be reduced into the other by fixing some parameters. Regression routines of modern languages for data analysis can furnish as output robust log-likelihood estimates of the regression model. By indicating with $\mathscr{L}_X$ the likelihood of the best estimate of model X, and by $\mathscr{L}_Y$ the likelihood of the best estimate of model Y nested in X, the ratio

$$\lambda = -2\ln\frac{\mathscr{L}_Y}{\mathscr{L}_X}$$

under certain conditions[9] is distributed as χ^2_f where the number of degrees of freedom $f = x - y$ with x, y being the number of parameters of X, Y. Thus, the p-value corresponding to the calculated λ allows to have an estimate of the likelihood that the our data are described just from the "restricted" model X. For the data analysed in the main paper, in the case of Nivi's and Metcalfe's models (the former can be reduced to the latter by costraining γ to be equal to 2) we find by the log-likelihood ratio test that in almost all the cases the null hypothesis $\gamma = 2$ corresponds to p-values less than 10^{-2}, i.e. it is extremely unlikely.

Information-theoretic approach to model selection

Like in log-likelihood ratio tests, model selection based on some form of statistical null hypothesis testing requires that one model is reducible to the other by constraining the parameter space. On the other hand, model selection based on information theory represents a quite different approach in the statistical sciences: while null hypothesis approaches are more suitable for classic experiments (control/treatment with randomization and replication), the information-theoretic approach is more suitable for the analysis of observational studies' data[10]. Starting from the concept of Kullback-Leibler distance $I(A,T)$ that measures the information lost when using a model A to approximate the real behavior T, Akaike[11,12] showed that "an information criterion" (AIC)

$$AIC = -2\log\mathscr{L} - 2K$$

estimates the expected, relative distance between the model fitted to the data and the unknown true process that actually generated the observations; here the bias-correction term K indicates the number of estimable parameters. In our analysis, since when the number of observations n is low (say $n/K < 40$) , we use the small sample AIC[13]

$$AIC_c = -2\log\mathscr{L} + \frac{2K(K+1)}{n-K-1}$$

that uses a modified biased correction term. To proceed to model selection, we compute the AIC differences

$$\Delta_i = AIC_i - AIC_{min}$$

over all the candidate models. Such differences estimate the *relative* expected K-L distances between the model an the "true" mechanism. The Δ_i allow to compare and rank candidate models; obviously, the best model has $\Delta_i = 0$. When the number of models to compare is small, some rough rules of thumb[10] are available:

Δ_i	Level of Empirical Support of Model i
$0-2$	Substantial
$4-7$	Considerably less
> 10	Essentially none

Notice that since the likelihood of a model given the data is $\propto \exp(-\Delta_i/2)$, Akaike advocated the use of Akaike's weights

$$w_i = -\frac{e^{\frac{\Delta_i}{2}}}{\sum_i e^{\frac{\Delta_i}{2}}}$$

. The Akaike weights not only provide an effective way to scale and interpret the Δ_i values, but also allow to calculate the evidence ratios w_i/w_j corresponding to the relative likelihood of occurrence of the model pairs i, j[10].

Models' predictive performance

To estimate the predictive performance of a model, an useful quantity is the mean squared prediction error measuring the accuracy of a model's prediction on a subset of data when the model's parameters are regressed to the remaining data. In particular, since our data sets contain a limited number of observations, we will use just the mean squared prediction error calculated via a leave-one-out (LOO) procedure, i.e. predicting the ith observation by fitting the remaining $n-1$.

In our case, for each model we calculate the $MSPE$[14]

$$MSPE = \frac{1}{n}\sum_i (V_i - \hat{V}_i)^2$$

where n is the size of the dataset, V_i is the ith observed network value (aka its advertising revenues), $\hat{V}_i$ is its estimate by fitting the model on all observation but the ith. Given a dataset, a model will have a better predictive performance (in our case, on single data) if it has a lower $MSPE$. Thus, to compare the predictive performance of the different models, we evaluate the ratio among the model's $MSPE$ respect to Metcalfe's model $MSPE$. Thus, a ratio < 1 indicates a better prediction performance respect Metcalfe's model, while a ratio > 1 indicates a worse performance.

References

1. Tongia, R. & Wilson III, E. J. The dark side of Metcalfe's Law: Multiple and growing costs of network exclusion. In *Beyond Broadband Access Workshop* (2010).
2. Kovarik, B. *Revolutions in communication: Media history from Gutenberg to the digital age* (Bloomsbury Publishing USA, 2015).
3. Briscoe, B., Odlyzko, A. & Tilly, B. Metcalfe's law is wrong-communications networks increase in value as they add members-but by how much? *IEEE Spectr.* **43**, 34–39 (2006). Publisher: IEEE.
4. Metcalfe, B. Metcalfe's law after 40 years of ethernet. *Computer* **46**, 26–31 (2013). Publisher: IEEE.
5. Nivi, B. Between Metcalfe's and Reed's laws (2005).
6. Reed, D. P. That sneaky exponential—Beyond Metcalfe's law to the power of community building. *Context. magazine* **2** (1999). Publisher: Spring.
7. Dunbar, R. I. Neocortex size as a constraint on group size in primates. *J. human evolution* **22**, 469–493 (1992). Publisher: Elsevier.
8. Odlyzko, A. & Tilly, B. A refutation of Metcalfe's Law and a better estimate for the value of networks and network interconnections (2005).
9. Li, B. & Babu, G. J. *A graduate course on statistical inference*. Springer texts in statistics (Springer, New York, NY, 2019).
10. Burnham, K. P., Anderson, D. R. & Burnham, K. P. *Model selection and multimodel inference: a practical information-theoretic approach* (Springer, New York, 2002), 2nd ed edn. OCLC: ocm48557578.
11. Akaike, H. Information theory as an extension of the maximum likelihood principle. á in: Petrov, bn and csaki, f. In *Second International Symposium on Information Theory. Akademiai Kiado, Budapest, pp. 276Á281* (1973).
12. Akaike, H. Information measures and model selection. *Int Stat Inst* **44**, 277–291 (1983).
13. Sugiura, N. Further analysts of the data by akaike's information criterion and the finite corrections: Further analysts of the data by akaike's. *Commun. Stat. Methods* **7**, 13–26 (1978).
14. Gareth, J., Daniela, W., Trevor, H. & Robert, T. *An introduction to statistical learning: with applications in R*. 103 (Spinger, 2013).

(US)	Reed	Nivi	Metcalfe	Odlyzko-Tilly	Sarnoff
Facebook	0.954	0.958	0.622	0.506	0.467
Instagram	0.994	0.992	0.710	0.532	0.461
YouTube	0.996	0.994	0.625	0.454	0.401
Google	0.940	0.927	0.709	0.551	0.492
Reddit	0.987	0.980	0.909	0.726	0.600
LinkedIn	0.977	0.963	0.869	0.691	0.588
Pinterest	0.854	0.863	0.631	0.450	0.379
Snapchat	0.963	0.955	0.666	0.512	0.444
Twitter	0.958	0.953	0.785	0.669	0.602

Table S1. US data: R^2 values for Reed's, Nivi's, Metcalfe, Sarnoff's and Odlyzko-Tilly's laws.

META	Reed	Nivi	Metcalfe	Odlyzko-Tilly	Sarnoff
World	0.977	0.995	0.990	0.875	0.836
US	0.993	0.991	0.785	0.620	0.567
Canada	0.951	0.977	0.910	0.751	0.628
UK	0.993	0.988	0.857	0.687	0.588
Italy	0.983	0.992	0.898	0.733	0.629
Spain	0.968	0.982	0.885	0.716	0.600
Germany	0.994	0.994	0.854	0.691	0.594
France	0.963	0.977	0.826	0.633	0.525
OtherCountries	0.975	0.985	0.982	0.872	0.830

Table S2. Meta data: R^2 values for Reed's, Nivi's, Metcalfe, Sarnoff's and Odlyzko-Tilly's laws over different countries.
